# Editorial: Role of Silicon in Plants

**DOI:** 10.3389/fpls.2017.01858

**Published:** 2017-10-25

**Authors:** Rupesh K. Deshmukh, Jian F. Ma, Richard R. Bélanger

**Affiliations:** ^1^Département de Phytologie–Faculté des Sciences de l'Agriculture et de l'Alimentation, Université Laval, Québec, QC, Canada; ^2^Group of Plant Stress Physiology, Institute of Plant Science and Resources, Okayama University, Kurashiki, Japan

**Keywords:** silicon uptake, abiotic stress tolerance, biotic stress tolerance, transport dynamics, physiology

Silicon (Si), the second most abundant element on earth surface, is rapidly gaining attention in agriculture because of its many beneficial effects for plants. Hundreds of studies performed with several plant species and under diverse growth conditions have demonstrated the favorable benefits of Si fertilization, particularly in alleviating biotic and abiotic stresses (Fauteux et al., 2005, 2006). Ever since the breakthrough discovery of genes explaining the molecular mechanisms of Si uptake and transport in plants a decade ago (Ma et al., 2006, 2007), many research endeavors have tried to explain how and why Si presence in plants confers advantages. The most challenging aspect consists in defining a mechanistic model explaining the precise mechanisms involved in Si-derived stress tolerance. While many hypotheses have been proposed, there is no conclusive evidence showing exactly how Si plays a role in stress tolerance. Current efforts to resolve this enigma involve comprehensive analyses of the effect of Si supplementation on various abiotic and biotic stresses, biochemical and physiological parameters, mineral co-localization and distribution, and transcriptomic and metabolomic responses. At the same time, research activities are focused on improving Si fertilization and Si sources for crop cultivation. The present research topic compiles many aspects helpful to generate a better understanding required for the optimal utilization of Si to promote sustainable development and climate-adapted cropping.

## Role of silicon in abiotic stress tolerance and plant physiology

Abiotic stress is one of the most severe constraints for crop cultivation all over the world. Because of climate change and unpredictable weather, abiotic stresses have become more common and challenging. Plants generate reactive oxygen species (ROS) as a first response to most abiotic stresses like salinity, drought, thermal, and heavy metal stress. This response is known to cause severe damages to cell structure and organelles, and to alter normal cell function. A study conducted by Hasanuzzaman et al. has shown that plants growing under heavy metal stress (cadmium) had reduced ROS contents when supplemented with Si compared to control plants. The improved antioxidant defense mechanisms against Cd stress with Si supplementation was found to be associated with an efficient augmentation of antioxidant components, associated with an increased activity of AsA-GSH and glyoxalase pathways. Similarly, Pontigo et al. also observed that Si-derived aluminum (Al) stress tolerance in ryegrass was associated with a change in ROS profile and reduced uptake of Al by plants from the soil.Incidentally, a review article by Kim et al. discusses the role of Si in abiotic stress and its possible implication in regulating the generation of ROS.

Abiotic stress significantly affects physiological processes leading to altered metabolic activities and overall health of plants. Grasses are well-known high accumulators of Si, and, therefore, serve as an excellent model to study the role of Si in plant physiology. To investigate the passive and active regulation of Si transport, McLarnon et al. evaluated some physiological parameters in three genotypes of forage grass differing in their ability to accumulate Si. Their results suggest that the varietal differences are attributed to stomatal conductance and transpiration, particularly when plants are grown under control conditions. However, under stress (wounding), an increased level of Si was noticed in all three genotypes, a reaction attributed to a higher expression of Si transporter genes. These results suggest an active mode of regulation of Si uptake under stress conditions. Similarly, Soundararajan et al. observed an improved stomatal development in tissue-cultured carnation plants supplemented with Si. This was correlated with a differential expression of proteins linked to photosynthesis, ribosomes, oxido-reduction, hormone signaling, metal ion binding, and defense responses. Manivannan and Ahn have critically reviewed several such studies suggesting a role of Si in regulating physiological processes in plants. Similarly, based on several studies conducted over the last decade,Rios et al. proposed a model explaining how Si could improve stomatal functioning and enhance root hydraulic conductance through the regulation of aquaporins. The effect of Si on the transcriptional regulation of genes involved in water transport and stress related pathways, including the jasmonic acid pathway, ABA-dependent or independent regulatory pathway, and phenylpropanoid pathway, have been proposed in several studies (Manivannan and Ahn; Rios et al.). Nevertheless, there is still no conclusive evidence for the direct active involvement of Si in any metabolic processes that can explain systematically how Si regulates cellular processes.

## Role of silicon in biotic stress tolerance

The beneficial effects of Si in improving tolerance against diseases and pests are arguably the most commonly described. A review article by Wang et al. provides a catalog of many significant studies and discusses models explaining the role of Si. For a long time, Si-derived resistance to pathogens and insects was thought to be the result of a mechanical barrier formed by the deposition of Si along the cell wall thus hindering their progression. However, studies performed in the 90's associated the presence of Si with specific defense responses *in planta* (Chérif et al., 1992, 1994; Fawe et al., 1998), a phenomenon that has since been shown in many host–pathogen interactions (Fauteux et al., 2005). In a recent study, Si was further shown to interfere with host–pathogen recognition, probably by preventing effectors and signaling molecules from finding their specific targets (Vivancos et al., 2015). Silicon was also suggested to induce indirect defense mechanisms by altering the composition of herbivore-induced plant volatiles (HIPV) (Liu et al.). The HIPV compounds play an important role in attracting parasitoids to infested rice plants. The list of studies reporting prophylactic effects of Si against diseases grow continuously but the underlying molecular mechanisms explaining these properties are not yet fully understood. Nevertheless, these studies offer additional support to explore Si as an environmental-friendly option for sustainable crop management.

The evaluation of different sources of Si is a critical aspect to optimize the practical use of Si fertilization. In this context, Ouellette et al. tested different Si fertilization regimes under high tunnel and field conditions for strawberry production. Under high tunnel, strawberry plants accumulated as much as 3% d.w. Si, which resulted in significant reduction of powdery mildew severity and higher yields. On the other hand, strawberry plants grown in soil, were unable to absorb Si, whether amended in liquid or solid form. Similarly, Keeping tested several sources of Si including fused magnesium (thermo) phosphate, volcanic rock dust, magnesium silicate, calcium silicate slag, and granular potassium silicate for sugarcane plant growth. Only the latter source led to a significant increase in Si accumulation. These studies suggest that Si sources and modes of application will greatly influence Si accumulation in different plant species. Therefore, more extensive efforts are required to better understand the relationship between Si sources and soil properties to obtain higher levels of plant-available Si. Apart from these conventional sources, nano-technological advances are also being used to explore possibilities for the application of Si nanoparticles as a source to elevate stress tolerance in plants (Luyckx et al.).

## Silicon dynamics and distribution in plants

Understanding the dynamics of molecular movement is essential to explain how plants take up water and mineral elements from the soil and subsequently distribute them to different tissues. While protein polarity and expression regulation of *Lsi1* and *Lsi2* transporter genes are well defined, less is known about investment efficiency and the functionality behind the regulation. A new model simulating the dynamics of Si in the whole rice plant has been developed by Sakurai et al. taking into account Si transport, distribution, and gene expression. Results of the simulation experiments suggest that rice has evolved a system maximizing the investment efficiency of Si uptake. Another study in rice by Hinrichs et al. has shown the involvement of an ATP binding cassette (ABC) transporter (OsABCG25) in the Si-promoted Casparian band formation. This study associates the processes of Si-promoted Casparian-band formation and the role of the exodermis with flux control in the roots.

Deposition of Si mostly occurs in leaf epidermal cells, in outer epidermal cells of inflorescence bracts and in root endodermis. While this influences passive mechanisms like transpiration, Si deposition is not a random process. In a review article, Kumar et al. describe Si depositions patterns, based mostly on observations in grasses, and suggest three dominant modes of Si deposition, namely directed paramural silicification in silica cells, spontaneous cell wall silicification, and directed cell wall silicification.

Silicon deposition has been mostly studied in root and aerial vegetative tissues, while very few efforts have been made to understand Si deposition in seeds. Bokor et al. offered a rare look in this field by exploiting ionomics to study Si in maize kernels. Ionomics results showed a significant correlation between Si deposition and other elements like Mg, P, S, N, P, Ca, Cl, Zn, and Fe. Bokor et al. observed Si accumulation in the pericarp and embryo but not in the soft endosperm or the scutellum. These studies describing Si dynamics and distribution in plants contribute new findings toward elucidating the role of Si *in planta*.

## Outlook

In spite of the ubiquitous presence and effect of Si on numerous plant species, Si research has been mostly conducted on monocots in general, and grasses in particular. This is evidently attributable to the fact that grasses are high Si accumulators, and that rice is a convenient and useful model for Si studies. However, many dicots and primitive plant species are equally known to accumulate high amounts of Si, and recent advents in genomics have confirmed a high level of conservation in Si transporters across the plant kingdom (Deshmukh and Bélanger, 2016). These new finding should stimulate Si research on different plant species and help unravel the complexities of Si properties in plants. Of particular interest, there is a need to dissociate Si from correlative roles, and design experiments aimed at defining with precision whether Si is metabolically or biochemically active or not. In the same manner, the prophylactic properties of Si against biotic and abiotic stress are more than likely linked to a universal phenomenon rather than a multitude of hypotheses, and collaborative efforts should seek to elucidate this mystery. Concerted efforts in Si research can only lead to its accelerated and improved application in the context of sustainable agriculture.

## Author contributions

All authors listed have made a substantial, direct and intellectual contribution to the work, and approved it for publication.

### Conflict of interest statement

The authors declare that the research was conducted in the absence of any commercial or financial relationships that could be construed as a potential conflict of interest. The handling Editor declared a past co-authorship with one of the authors JM.
